# EGFR-Based Immunoisolation as a Recovery Target for Low-EpCAM CTC Subpopulation

**DOI:** 10.1371/journal.pone.0163705

**Published:** 2016-10-06

**Authors:** Ana Vila, Miguel Abal, Laura Muinelo-Romay, Carlos Rodriguez-Abreu, José Rivas, Rafael López-López, Clotilde Costa

**Affiliations:** 1 INL–International Iberian Nanotechnology Laboratory, AvdaMestre José Veiga, 4715–330 Braga, Portugal; 2 Translational Medical Oncology, Health Research Institute of Santiago (IDIS); ComplexoHospitalarioUniversitario de Santiago de Compostela (SERGAS); Trav. Choupana s/n 15706, Santiago de Compostela, Spain; 3 Liquid Biopsy Analysis Unit, Health Research Institute of Santiago (IDIS); ComplexoHospitalarioUniversitario de Santiago de Compostela (SERGAS); Trav. Choupana s/n 15706, Santiago de Compostela, Spain; 4 Department of Applied Physics, Technological Research Institute, Nanotechnology and Magnetism Lab—NANOMAG, University of Santiago de Compostela, 15782, Santiago de Compostela, Spain; Seoul National University College of Pharmacy, REPUBLIC OF KOREA

## Abstract

Circulating tumour cells (CTCs) play a key role in the metastasis process, as they are responsible for micrometastasis and are a valuable tool for monitoring patients in real-time. Moreover, efforts to develop new strategies for CTCs isolation and characterisation, and the translation of CTCs into clinical practice needs to overcome the limitation associated with the sole use of Epithelial Cell Adhesion Molecule (EpCAM) expression to purify this tumour cell subpopulation. CTCs are rare events in the blood of patients and are believed to represent the epithelial population from a primary tumour of epithelial origin, thus EpCAM immunoisolation is considered an appropriate strategy. The controversy stems from the impact that the more aggressive mesenchymal tumour phenotypes might have on the whole CTC population. In this work, we first characterised a panel of cell lines representative of tumour heterogeneity, confirming the existence of tumour cell subpopulations with restricted epithelial features and supporting the limitations of EpCAM-based technologies. We next developed customised polystyrene magnetic beads coated with antibodies to efficiently isolate the phenotypically different subpopulations of CTCs from the peripheral blood mononuclear cells (PBMCs) of patients with metastatic cancer. Besides EpCAM, we propose Epidermal Growth Factor Receptor (EGFR) as an additional isolation marker for efficient CTCs detection.

## Introduction

Metastasis remains the main cause of cancer-related deaths, dissemination through the blood circulation being the frontier between favourable localised and unfavourable systemic disease[1].Circulating tumour cells (CTCs) are tumour cells shed from an existing primary tumour or from metastatic lesions that circulate in the peripheral blood of patients with solid malignancies[2]. The isolation of CTCs presents a significant challenge because: i) CTCs are rare events in blood (the estimation is just 1 CTC per ~10^7^ white blood cells per millilitre of blood); ii) the blood volume available for CTCs detection in the clinical routine is limited (7.5 mL blood); iii) there are no CTC-specific or universal markers. Although many advances have been made regarding the detection and molecular characterisation of CTCs, several challenges still exist precluding the clinical use of CTCs in early detection and their characterisation as an important tool to monitor and prevent the development of overt metastatic disease [3].

CTCs have developed several mechanisms to survive in the blood andreach distant organs. They can escape anoikis, travelling with blood cellsand forming aggregates. Moreover, to reach the blood circulation,CTCs undergoan epithelial-to-mesenchymal transition process (EMT) and mesenchymal-to-epithelial transition (MET), giving rise to thewide variety of CTC phenotypes that have been described in the bloodstream. Multiple isolation techniques have been developed in recent years[3, 4], the CellSearch^®^system being the only one cleared by the FDA for clinical use in patients with breast, colon and prostate cancer. CellSearch^®^only enumerates epithelial phenotype CTCs (CD45-, EpCAM+ and cytokeratins 8, 18 and/or 19+) in whole blood. CTCs are isolated magnetically based on EpCAM expression and subsequent immunofluorescence for cytokeratins and DAPI, discarding CD45+ cells,which allows the identification of CTCs always taking into account strict morphologic criteria. Nevertheless, CellSearch^®^ only detects a sufficient number of CTCs for clinical purposes in 40–50% of patients with disseminated carcinomas and is not indicated for all tumour types[5, 6]. Many other strategies for CTCs isolation have been proposed in recent years such as size exclusion or microfluidic devices; although much progress has been done in this field, there is no clinical validationandCTC isolation based onEpCAM expressionremains the standard[3, 7].

In carcinomas, the EpCAM expression pattern changes to intense membranous overexpression with cytoplasmic staining [8, 9]. During dissemination, epithelial tumour cells undergo profile changes to overcome intravasation, to survive in the bloodstream and to form secondary tumours. Due to EMT, some cells could lose theirEpCAM expression although they can express it again at the metastasis site during the MET process[10, 11]. In addition, there is a reduction of cell-cell adhesion and loss of apical-basolateral polarity. If at least a subset of CTCs undergoes EMT, whereby epithelial markers are downregulated, technologies reliant on EpCAM expression for CTC capture might fail to enrich an important subpopulation of cells. In fact,CTCs can express or co-express epithelial, mesenchymal or stemness markers. Although CTCs are of epithelial origin, the main feature of cells that are able to metastasise is overcoming the EMT process where each CTC has its own identity and could represent a different CTC subpopulation. Thus, other markers are needed for the isolation of CTCs from patients with cancer [12, 13]. Importantly, if different CTCs subpopulations could be separated, it would be useful for determiningspecific progression and invasion patterns in the metastasis process, each one with distinct clinical significance.

Here we emphasise thatthe idea that the isolation of CTCs based solely on EpCAM expression is limited, as CTCs with low or no EpCAM expression would be omittedduring isolation. Therefore, we have designed magnetic beads that can be coated with different antibodies whichrecognise antigens highly expressed in diversetumour types and phenotypes. As a novelty, we propose a multistep isolation method using customised magnetic beads which includes in addition toEpCAM other markers as Epidermal Growth Factor Receptor (EGFR) and Fibroblast Growth Factor Receptor (FGFR) for the isolation of CTCs from the blood samples of patients with metastatic colon, prostate, breast and endometrial cancer.

## Methods

### Patients and samples

Patients participating in the study were recruited at the University Complex Hospital of Santiago de Compostela (Santiago de Compostela, Spain). The ethical committee Autonomy Committee on Research Ethics (CAEI from the Spanish acronym *Comité Autonómico de Ética de Investigación*) of Galicia with the code CAEI 2014/126approved the study and the consent procedure. It was signed by all patients whom previously were provided with written and verbal information to participate in this study. Samples processing was carried out in accordance with the approved guidelines. Two 7.5 mL samples were collected from peripheral blood of patients with metastatic cancer in a CellSearch^®^ tube (Veridex, LLC, Raritan) and a EDTA tube. Peripheral blood mononuclear cells (PBMCs) were isolated from the EDTA tubes by Ficoll density gradient centrifugation (Sigma-Aldrich, St. Louis, MO, USA) according to the manufacturer’s protocol; cell pellets were resuspended in 400 μL PBS-2% BSA- .05% Tween® 20.

The CellSearch^®^ analysis (Veridex, LLC) was performed by the Liquid Biopsy analysis Unit (Health Research Institute of Santiago, Spain) as described previously [14, 15],

### Cell lines

The following tumour cell lines were purchased from ECACC directly from Sigma-Aldrich:breast: MDA-MB-231[16](92020424), MCF7[17] (86012803); colon: HT29[18] (91072201), SW480[18] (87092801), SW620[18] (87051203); lung: A549[19] (86012804) and prostate: PC-3[20] (90112714); as well as endothelial cells: HUVEC (S200-05N) and leucocytes: Jurkat cells[21] (88042803).The brest cancer tumour cell HCC1937 lines[22](ATCC-CCL-247)and HCC1954[23] (ATCC CRL-2338) were purchased from ATCC; the endometrial cell lines HEC1A and HEC1A stably expressing the ETV5 transcription factor (HEC1A-ETV5) were previously described [24–26]. Medium for cell culture were obtained from Gibco (DMEM: SW480, SW620, A549, MCF7, MDA-MB-231, PC-3; McCoyy᾿s: HT29, HEC1A, HEC1A-ETV5; RPMI 1640: HCC1937, HCC1954,Jurkat), and from Lonza (Basel, Switzerland) (EGM-2: Huvec). The medium was supplemented with 10% FBS and 1% streptavidin-penicillin (Life Technologies, Carlsbad Ca, USA). Cells were maintained at 37°C in a 5% CO_2_ incubator. For selected experiments, we transduced MDA-MB-231 and SW480 cells with lentiviral transduction particles (turboGFP, Sigma-Aldrich) to establish cells that expressed green fluorescent protein (GFP); cells were further selected with puromycin (5 μg/mL) and checked by flow cytometry (96%).

### Flow cytometry

Cells were kept in culture for 72 hours. Cells collected for flow cytometry were washed with 1x PBS, trypsinised, and resuspended in PBS-2% BSA-0.05% Tween® 20. One million (1 x 10^6^) cells were used for each labelling. The primary antibodies used were: anti-CD49f-PE (BD, Pharmigen, Franklin Lakes, NJ, USA); anti-EpCAM, anti-MUC1, anti-EGFR, anti-N-cadherin, anti-E-cadherin, anti-CD66a/c/e (Biolegend, San Diego, CA, USA); anti-EpHB4 and anti-claudin-3 (R&D Systems, Inc., MN, USA); anti-vimentin, anti-claudin-4, anti-ALDH1, anti-FGFR; secondary antibodies were Alexa488 and Alexa647 (Abcam, Cambridge, UK). Unlabelled cells and with only secondary antibody labelling were included in each independent assay and considered autofluorescent. Dead cells and debris were excluded from the analysis. Cells were analyzed in a FACs-Aria flow cytometer (BD Bioscience).

### Immunofluorescence assay

Immunofluorescence analyses were performed using cells fixed with 4% paraformaldehyde. The antibodies used were anti-pan-cytokeratin (4, 5, 6, 8, 10, 13, 18)-Alexa 488 (Exbio) and anti-CD45-PE (Exbio). Samples were incubated with Hoechst (Sigma- Aldrich) for nuclear staining and were visualisedunder a fluorescent microscope (AxioVertA1, Zeiss).

### RNA extraction and quantitative real-time PCR (RT-qPCR)

RNA purification was performed with a Qiamp Viral kit (Qiagen, Valencia, CA, USA) optimised for very low-cellularity samples. cDNA was synthesised using Super ScriptIII chemistry (Invitrogen, Carlsbad, CA, USA) according to the user’s guide and subjected to pre-amplification with a TaqMan® PreAmp Master Mix kit (Applied Biosystems, Foster City, CA, USA) for 14 reaction cycles before proceeding to RT-qPCR. To measure the gene expression levels in CTCs isolated from EpCAM, EGFR and FGFR fractions, TaqMan Gene Expression Assays (Applied Biosystems) were used for 7 selected genes (E-cadherin, vimentin, *ALDH1*, *PROM1*, *ZEB2*, *SNAIL* and *CD45* as a marker of non-specific isolation). Values were analysed using StepOne Software v.2.1 (Applied Biosystems), normalised to *CD45* and represented as (40–ΔCt), whereby ΔCt = duplicate mean (CtTARGET–Ct*CD45*).

### Preparation and characterisation of magnetic polymer beads

First, oleic acid capped magnetite nanoparticles (OMN) were prepared by precipitation from two iron salts (FeCl_3_ and FeCl_2_) in the presence of ammonium hydroxide followed by the addition of oleic acid [27]. The OMN average size is 10 nm with a polydispersity index of 0.2 as determined by Transmission Electron Microscopy (TEM; TITAN® 200 Kv ChemiS TEM) and Dynamic Light Scattering (DLS) cumulant analysis (Horiba Scientific SZ-100) [28].

Polymer beads loaded with magnetite nanoparticles were prepared by mini-emulsion polymerisation. Aliquots of OMN stock suspension in cyclohexane were first dried and then homogeneously dispersed in a mixture of styrene (Styr), divinylbenzene (DVB), methacrylic acid (MA) and the polymerisation initiator azobisisovaleronitrile (ADVN) (mass ratio Styr: DVB:MA:ADVN = 76:13:10:1). This mixture was emulsified by sonication (Branson® Digital Sonifier® S250D, 2 min at 10% power) in an aqueous sodium dodecyl sulfate solution (SDS 2.5 wt%) and then the polymerisation process was carried out at 70°C for 18–24 h.

Beads with different magnetite content were obtained and characterised. Magnetite content was determined by thermogravimetric analysis (TGA/DSC1 STAR systems, Melter Toledo); average size was determined by DLS and confirmed by scanning electron microscopy (SEM; Quanta ESEM); magnetic parameters were measured using a Vibrating Sample Magnetometer (VSM, EV9; Micro-Sense), yield (wt%) was determined by weighing dried sediments (previous supernatant removal after magnetic isolation).

Polymer beads were prepared with SDS as a surfactant, which is very harsh on cells, and its elimination is required for cell studies. Nevertheless, complete removal of surfactant could compromise bead colloidal stability and cause the formation of aggregates. Taking this into account, several mediums compatible with viable cells and colloidally stable beads were tested for handling, of which Tween® 20 solution (0.05 w-t%) was found to be the best. Polymer beads were washed up to 4 times (using magnetic separation) and the size was measured. In addition, the colloidal stability of surfactant-formulated polymer beads during storage (at room temperature) was monitored for 16 months.

### Functionalisation of magnetic polymer beads

Once the polymer beads were prepared, the next step was to functionalize them and study their interaction with EpCAM-, EGFR- and FGFR-expressing cancer cells.

The carboxylic groups of MA on the surface of the magnetic polymer beads were activated using two reagents:1-ethyl-3-[3-dimethylaminopropyl] carbodiimide (EDC) and N-hydroxysulfosuccinimide (Sulfo-NHS) in the presence of Tween® 20 (as a dispersing agent) for 15 min at room temperature. After incubation, these reagents were removed very quickly (centrifugation at 22000 rpm for 10 min) in order to avoid bead aggregation. This activation enables reaction with primary amines to form amide bonds. The activated magnetic polymer beads were incubated with proteins (protein A or albumin) for 3 h at 4°C to allow binding of the proteins to the surface of the beads.

After protein A coating, the beads were incubated with the following selected antibodies (of mouse origin): anti-EpCAM (Biolegend), anti-EGFR (Dianova, Hamburg, Germany) and anti-FGFR (Abcam) which were efficiently attached to the surface of the beads for the cell studies.

### Isolation assay

In each independent assay we spiked cells in incubation buffer (PBS-2% BSA-0.05% Tween® 20) and mixed them with magnetic beads for 1 h at 4°C under orbital rotation. Isolation was carried out with a magnet (Life Technologies) for 5 min. Recovery was measured in a haemocytometer (BlauBrand). The percentage is calculated taking into account the number of spiked cell and the number of isolated cells. When the initial number of spiked cells was low (under the detection limit of the haemocytometer), GFP-positive cells were used and counted by scouring dish under a fluorescence microscope AxioVertA1 (Carl Zeiss, Jena, Germany).

### Cell Viability assay

To determine cell proliferation of cells isolated with magnetic beads, AlamarBlue® (Life Technologies) was added to cells in a 96-well plate for 3 h at 37°C, 5% CO_2_ (1:10 final dilution in culture medium). Fluorescence was read on a FLUOStar Optima (BMG Labtech, Germany).

### Statistical analysis

Data are expressed as mean ± standard deviation (SD). The statistical analysis of the data was performed by using the statistical analysis software GraphPad Prism, version 6.01. The differences between MDA-MB-231 cells yielded using EpCAM- or EGFR-coated beads were analysed using the two-tailed Student’s *t*-test. Comparisons within total CTCs isolated from patients with metastatic cancer and CTCs counted by CellSearch® and gene expression analysis were assessed using the Wilcoxon signed rank test(95% confidence intervals). Correlation analysis was performed using Spearman᾿s rank correlation coefficient. Kaplan–Meier method was used for survival analysis. Findings of *p* < 0.05 were considered significant.

## Results

### Characterisation of tumour cell lines

We first screened a large panel of markers, including EpCAM, for more effective isolation of CTCs from the peripheral blood of patients with metastatic cancer. We selected a panel of11 human tumour cell lines of 5 distinct tumour type origins, displaying cellular phenotypes representative of the heterogeneity of tumour cell populations(Table 1). This panel included colon tumour cell lines (SW480, SW620 and HT29); a lung tumor cell line (A549);breast tumour cell lines (MCF7, HCC1937, HCC1954 and MDA-MB-231); endometrial tumour cell lines (HEC1A and HEC1A-ETV5) anda prostate tumor cell line (PC-3).These tumour cell lines are all of epithelial origin; however, the SW620, MDA-MB-231 and PC-3 cell lines are from metastatic sites and have a more mesenchymal-like phenotype together with A549 and HEC1A-ETV5 cells[26, 29–32] (S1 Table). We next screened by flow cytometry this panel of cell tumour phenotypes with epithelial markers (EpCAM, E-cadherin, Muc1, claudin-3 andclaudin-4) or non-epithelial markers (N-cadherin, vimentin, CD66, EGFR, FGFR, EphB4,ALDH1 and CD49f). We further included an endothelial cell line and a lymphocyte cell line as negative controls. As shown in Table 1,vimentin, CD49f and claudin-3 were expressed in endothelial or lymphocyte cells, and were thus discarded for further characterisation of tumour cell phenotypes. N-cadherin, claudin-4, CD66, FGFR and ALDH1 demonstrated residual expression in the vast majority of the evaluated cell lines and/or suboptimal labelling in individual lines (Table 1). Similarly, EphB4 was expressed at significant levels in MDA-MB-231, HCC1937, A549 and HT29 cells, while Muc1 was present at high levels only in SW480 cells (Table 1). These markers did not demonstrate utility for the efficient isolation of the general heterogenic tumour cell populations examined in this study.

**Table 1:** Summary of flow cytometry analysis.

	EpCAM	E-cadherin	Muc1	Claudin-3	Claudin-4	N-cadherin	Vimentin	CD66	EGFR	FGFR	EphB4	ALDH1	CD49f
**SW480**	94.50	57.90	97.80	32.80	1.22	0.79	0.40	37.70	51.90	9.04	0.66	6.87	99.50
**SW620**	60.00	9.21	15.50	28.40	3.61	0.15	3.56	4.84	0.22	2.10	0.07	0.16	99.90
**HT29**	99.50	86.50	0.85	87.90	12.90	0.58	1.02	37.40	69.10	0.58	96.40	10.31	99.20
**A549**	38.70	7.95	5.65	58.00	18.40	0.26	7.92	9.21	93.30	0.30	55.90	11.80	99.80
**MCF7**	94.25	5.35	44.00	87.40	10.20	0.43	9.21	33.50	2.79	2.61	44.10	17.20	66.60
**HCC1937**	99.50	58.05	42.10	27.80	5.83	0.29	3.12	38.30	93.80	0.07	61.30	19.10	87.50
**HCC1954**	99.40	66.50	15.10	0.31	0.50	0.44	9.41	0.33	0.22	0.15	2.11	0.40	98.00
**MDA-MB-231**	5.47	21.00	20.50	79.70	13.10	0.21	18.30	1.07	92.57	3.22	94.40	92.30	93.70
**HEC1A**	92.30	90.80	2.28	9.59	1.70	1.23	2.50	0.77	97.10	12.70	0.10	9.76	98.60
**HEC1A-ETV5**	93.30	75.80	0.65	48.70	0.01	51.50	1.92	15.20	95.00	2.86	12.80	3.23	98.50
**PC3**	79.10	10.80	14.40	91.90	4.06	0.39	1.50	13.20	87.80	2.03	3.02	9.75	99.50
**HUVEC**	1.09	5.30	0.42	3.40	0.34	1.70	0.21	0.11	0.29	2.23	0.29	2.33	99.90
**Jurkat**	1.12	0.34	1.20	36.00	0.15	0.98	86.00	0.10	0.20	0.09	0.45	0.03	29.50

Listed proteins were analysedby flow cytometry in different tumour cell lines (colon: SW480, SW620, HT29; lung: A549; breast: MCF7, HCC1954, HCC1937, MDA-MB-231; endometrial: HEC1A, HEC1A-ETV5; prostate: PC-3; endothelial cell line: HUVEC and a lymphocyte cell line:Jurkat. Data represent % of cells with positive expression measured in 10000 cells per assay.

On the contrary, EpCAM and EGFR were found to be expressed at high rates (high percentage) and with moderate to high Median Fluorescence Intensity (MFI; S1 Fig) in most of the analysed tumour cell lines. Furthermore, the combination of these two markers demonstrates optimal complementarity for the efficient coverage of the different tumour cell phenotypes represented in the whole panel of 11 human tumour cell lines (Table 1). The cell lines expressing low levels of EGFR expressed EpCAM at high percentages and vice versa: A549 (EpCAM, 38.70%; EGFR, 93.30%), MCF7 (EpCAM, 94.25%; EGFR, 2.79%), HCC1954 (EpCAM, 99.40%; EGFR, 0%), and MDA-MB-231 (EpCAM, 5.47%; EGFR, 92.57%). We thus concluded that EGFR could complement EpCAM for CTC isolation, so we selected these two markers for the preparation of the immunomagnetic beads and the subsequent CTCs purification studies. Moreover, to further validate the utility of EGFR and EpCAM for efficient CTCs isolation, we explored FGFR as an example of a marker expressed at low percentages in almost all tumour cell lines checked, which could represent a minority tumour subpopulation.

### Preparation, characterisation and stability of magnetic polymer beads

**Fig 1:**
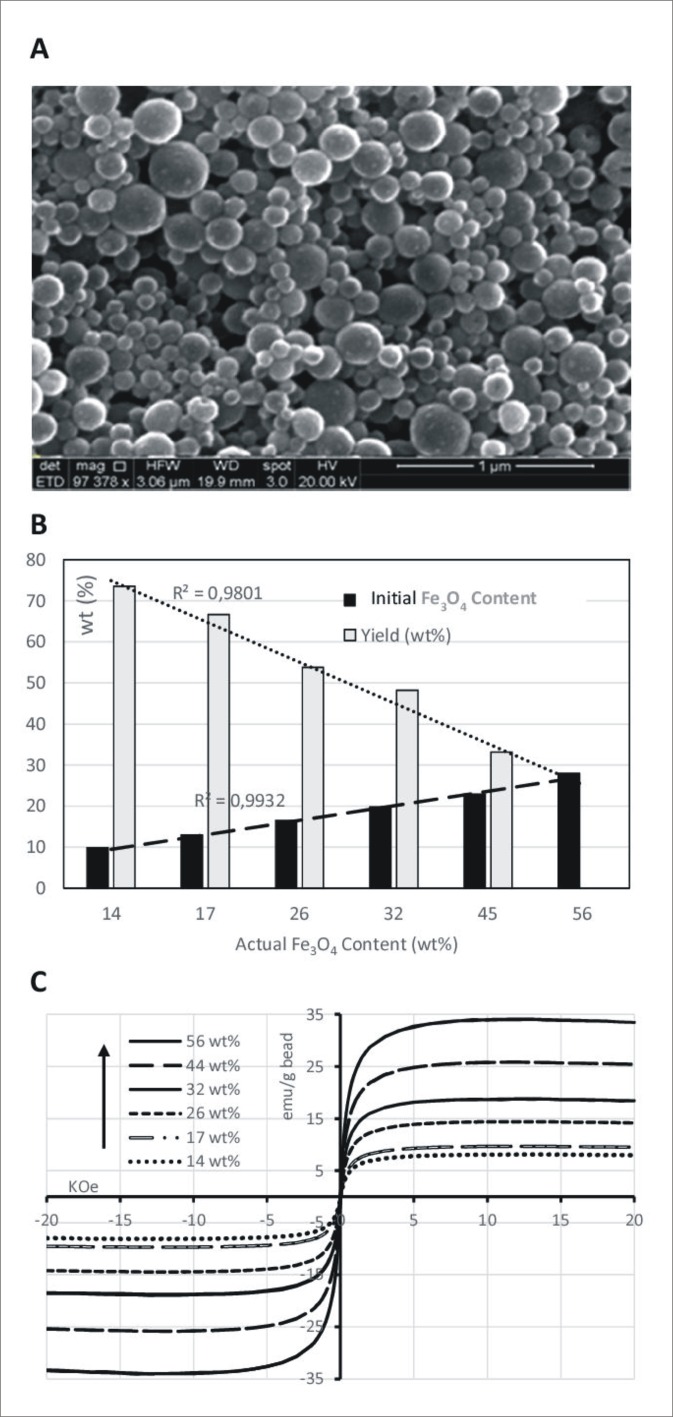
**A)** SEM image. **B)** Yield and initial Fe_3_O_4_ content versus actual Fe_3_O_4_ content (wt%). **C)** Plot of magnetisation versus applied magnetic field for beads.

The size of the synthesised beads (with different Fe_3_O_4_ content) ranged between 125 and 190 nm, as determined by SEM (Table 2; Fig 1A). In all prepared formulations, the final Fe_3_O_4_ content was higher than that estimated from the initial composition, which is attributed to the dissolution of MA in the aqueous phase and to incomplete polymerisation of Styr and DVB. There was good linear correlation (r^2^ = 0.9801) between the initial loading and the actual final loading (Fig 1B). Furthermore, higher initial Fe_3_O_4_ content leads to lower yields, i.e. the polymerisation process is less efficient and a higher amount of monomers remains dispersed in the aqueous phase and is discarded during washing and magnetic isolation.

**Table 2:** Characteristics of magnetic beads, including Fe_3_O_4_ content (TGA,wt%); average diameter (SEM); Yield(wt%) and saturation magnetization (Ms).

Initial Fe_3_O_4_ Content (wt,%)	Actual Fe_3_O_4_ Content TGA (%)	AverageSizebySEM (nm)	Yield (wt,%)	Ms (emu/g beads)
28	56	148	32	34.09
23	44	142	33	25.80
20	32	176	48	18.81
16	26	130	54	14.50
13	17	126	67	9.51
10	14	187	73	8.09

The saturation magnetisation (Ms) of the magnetic beads was proportional to the actual Fe_3_O_4_ content. Furthermore, all of the magnetic particles were in a superparamagnetic state and no hysteresis was observed; i.e. both remanence and coercive force values were zero, which is favourable to avoid aggregation of the assembly [33] (Fig 1C).

**Fig 2:**
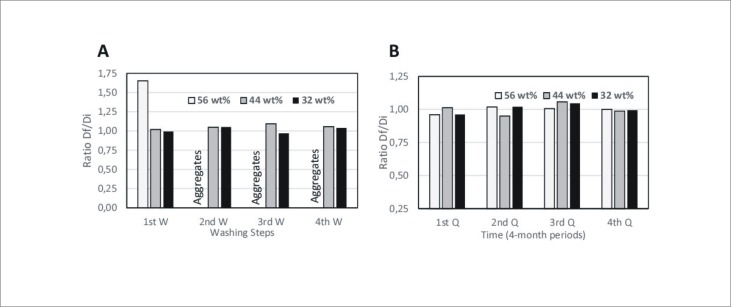
**A)** Df/Di (final diameter/initial diameter) ratio after several washing steps of magnetic beads. **B)** Df/Di ratio of beads after serial 4 months storage period.

The colloidal stability of magnetic polymer beads with the highest Fe_3_O_4_ content (52 wt%, 44 wt%, and 32 wt%) was tested in Tween® 20 solution (0.05 wt%). The magnetic polymer beads were washed four times and their size was measured initially and after each washing step. In Fig 2A the results are reported as the ratio of the bead diameter after each washing (Df) step divided by the initial diameter (Di), namely, (Df/Di). Beads with 44 wt% or 32 wt% were stable along all washing steps (Fig 2A). Taking into account these results, the sample of beads with 44 wt% Fe_3_O_4_ content was chosen for functionalisation and subsequent studies with CTCs. Batches with different Fe_3_O_4_ content (52 wt%, 44 wt% and 32 wt%) were stable for at least 16 months under storage at room temperature, showing no aggregation and retaining their functionalisation capability (Fig 2B).

In general, after the functionalisation process around 68–80% of the surface of polymer beads was covered by protein (determined by indirect quantification in supernatant using fluorescence or colorimetric methods) [28].

### Magnetic beads for cell isolation

#### Isolation of stable tumour cells using magnetic beads coated with antibodies

**Fig 3:**
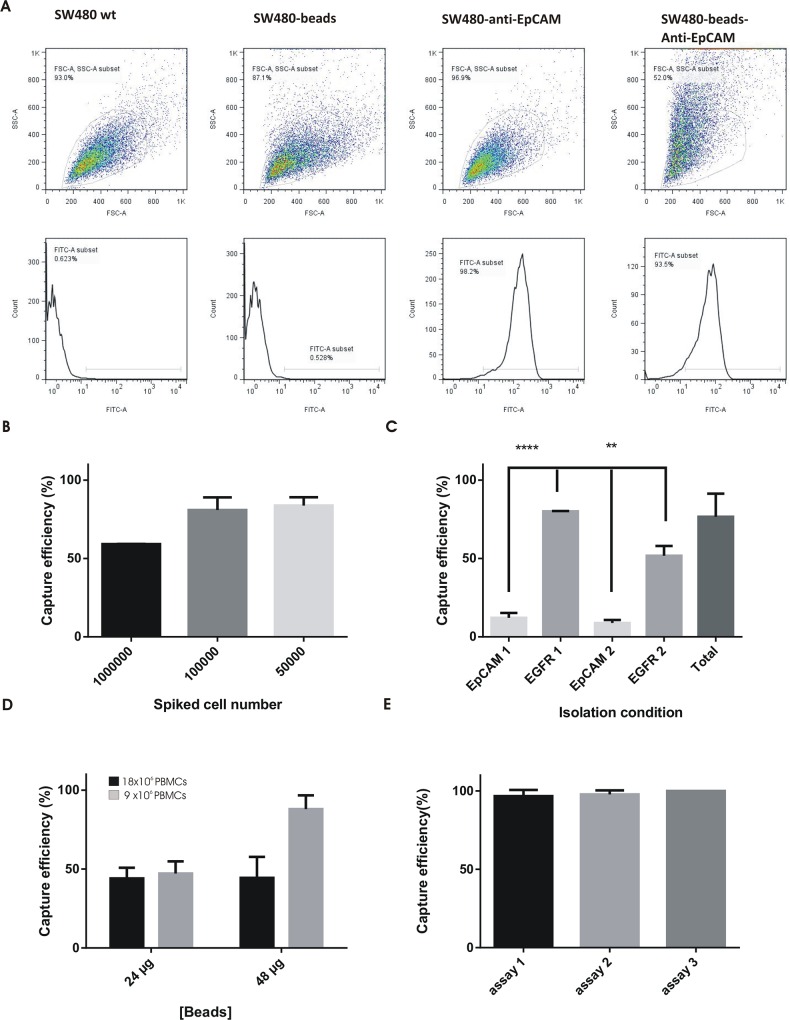
Cell recovery using magnetic beads. **A)** Flow cytometry analysis of SW480 cells incubated with anti-EpCAM antibody, beads coated with anti-EpCAM or magnetic beads alone. **B)** Capture efficiency (%) of SW480 cells with magnetic beads functionalised with anti-EpCAM (40 μg/μL) at different cell numbers. **C)** Capture efficiency (%) of MDA-MB-231 cells (10000) using anti-EpCAM beads or anti-EGFR beads (40 μg). Isolation was performed sequentially: EpCAM-EGFR or EGFR-EpCAM (1 or 2 indicates first or second isolation step, respectively). **D)** Capture efficiency (%) of SW480 cells (23.25±5.08) spiked in donor control PBMCs (9 or 18 million) for different amounts of anti-EpCAM beads (24 and 48 μg). Independent isolation assays, n≥3, (duplicates per condition). E) Capture efficiency (%) of SW480 cells (22.81±15.40) spiked in donor control PBMCs using 5.8 μg anti-EpCAM beads per million. Three independent isolation assays (n≥3 replicates per assay).

Once prepared, the specificity of magnetic beads coated with anti-EpCAM antibody waschecked in an EpCAM-positive cell line (SW480). For that, we performed a flow cytometry assay with anti-EpCAM magnetic beads or antibody alone using SW480 tumour cells. Anti-EpCAM magnetic beads labelled 93% of cells compared to 98% of cellbinding of anti-EpCAM alone (Fig 3A), demonstrating the high binding specificity of the beads to EpCAM-positive cells.

We next optimised the appropriate concentration of beads for magnetic separation by testing a concentration range of anti-EpCAM magnetic beads in SW480 colon tumour cells. For this, increasing numbers of cells were incubated with 40 μg beads following the same isolation protocol (timings and volumes). A higher beads/cells ratio led to a higher recovery percentage, with isolation efficiencies of 83.75%, 80.88% and 59.12% for 0.05, 0.1 and 1 million cells, respectively (Fig 3B). Using 85–95% as our reference in isolation efficiency, the adequate amount of beads per cell in PBS-2% BSA was very low, about 8-10x10^-4^μg.

To check if EGFR is a useful marker for cell isolation to compensate for the EpCAM limitation, we performed a magnetic isolation assay using anti-EGFR magnetic beads to compare the isolation efficiency to anti-EpCAM beads at the previous defined dose. For that, we selected a breast cancer cell line with low EpCAM expression (MDA-MB-231). Isolation with anti-EpCAM magnetic beads in MDA-MB-231 cells resulted in only 10% recovery while isolation with anti-EGFR magnetic beads enhanced recovery by up to 80%; regardless the order of isolation, anti-EGFR beads isolated significantly more cells than EpCAM for lowEpCAM expressing cell phenotypes (Fig 3C). Moreover, additional markers for cell isolation improved the yield and different fractions of isolated cells could represent distinct CTCs phenotypes.

Magnetic beads coated with anti-EpCAM or anti-EGFR could isolate tumour cells with high efficiency. To eventually use these beads in patient samples for CTCs isolation, we performed a virtual scenario to determine the most suitable amounts of beads in the presence of blood cells. It is important to take into account the number of PBMCs in samples because there is nonspecific bead binding to such cells (this behaviour was also observed with anti-EpCAM magnetic beads used in the CellSearch^®^ system and others; data not shown). For that, SW480 tumour cells were spiked in PBMC samples from healthy donors. To distinguish spiked cells from blood cells, the SW480 cell line was modified with a GFP. The number of spiked cells was 23 ± 5 per sample (9x10^6^ or 18x10^6^ PBMCs). Fig 3D shows the isolation efficiency using two amounts of beads (24 μg and 48 μg), which correlated inversely with that white blood cells. The best yield (90%) was obtained in spiked SW480 cell samples with 9x10^6^ PBMCs using 48 μg of beads. This study permitted us to determine that 5.8 μg is an adequate amount of beads per million PBMCs for use in sample patients. To test the beads dose we performed 3 independent assays of spiked tumour cells (SW480) in healthy blood using 5.8 μg per million PBMCs (medium capture efficiency [%] 98.26 ± 2.70) (Fig 3E). In addition, this concentration of beads (5.8 μg/million PBMC) allowed us to perform posterior validation by immunofluorescence without any interference.

#### Isolation of CTCs using magnetic beads retains cells viability

**Fig 4:**
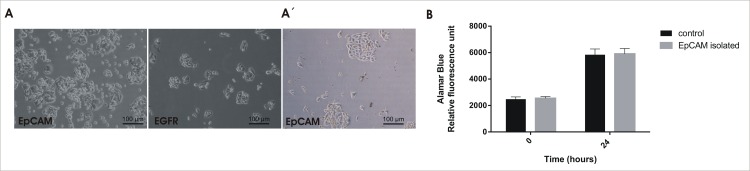
Viability of captured tumour cells. **A, A´)** Microscopic images of spiked SW480 cells isolated from PBS (A) or healthy donor PBMCs (A´). **B)** Cell proliferation was measured by Alamar Blue assay after 24 h in culture. Each point represents the mean ± S.E.M. of 6 replicates per group.

To analyse the toxicity of magnetic beads during the immunoisolation of CTCs, we assessed the viability of purified cells in culture (Fig 4A). Tumour cells coated with beads were able to grow in culture as non-isolated wild type cells, reached confluence and could be unlimitedly plated. It must be taken into account that each cell division leads to a loss in beads burden, resembling the wild type phenotype more closely.

Microscopy analysis showed no phenotype differences between magnetic beadsisolated cells and non-isolated wild type cells. Although the uptake of magnetic beads by cells was proven due to their small size, no impact on the proliferation cell rate was observed (Fig 4B).

These data confirm that the proposed protocol for CTCs isolation does not affect cell viability, encouraging further steps regarding CTCs culturing and characterisation. We confirmed that different tumour cell lines (SW480, MDA-MB-231, MCF7, A549) isolated with magnetic beads were able to grow in culture (data not shown), further demonstrating the universality of the CTCisolation protocol independently of tumour cell line type. Tumour cells isolated with magnetic beads from PBMCs from healthy donors (previously spiked) were able to grow in culture as well (data not shown).

#### Magnetic beads for CTCs isolation from blood of patients with cancer

**Fig 5:**
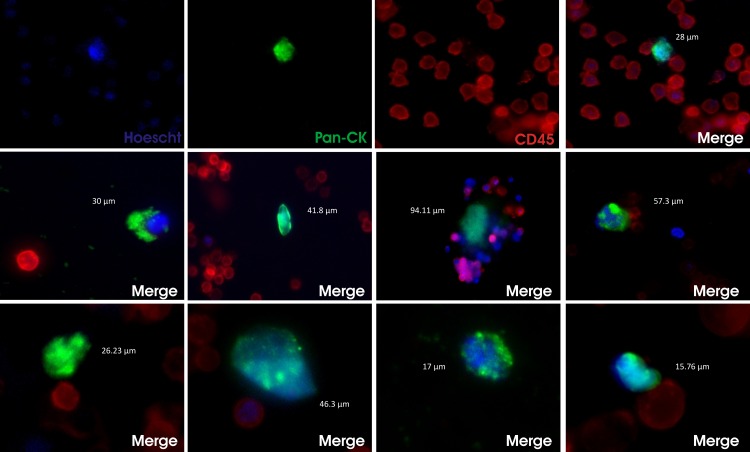
CTCs isolated with magnetic beads from blood from patients with metastatic cancer. Representative immunofluorescence for Hoechst (blue, for nuclei staining), pan-cytokeratins (green), CD45 (red) and merge of CTC isolated from a patient with metastatic breast cancer (upper panel). Merge for indicated staining of CTCs isolated with anti-EpCAM, anti-EGFR or anti-FGFR magnetic beads. The size of each CTC is indicated in μm.

Finally, to validate our approach in patients with metastatic cancer we isolated PBMCs from a cohort of 22 patients with colon (n = 7), prostate (n = 4), breast (n = 4) and endometrial (n = 7) cancer and disseminated disease. Samples were incubated as previously described with 5.8 μg beads per million PBMCs for each patient (16.01 ± 12.20 million PBMCs). The observed variability on the PBMCs number per patient was dependent on the healthy state of the patient due to the tumor burden or the chemotherapy treatment. The dose was selected taking into account the previous study (beads per million PBMC) and 10% extra (due to the variability in the number of CTCs). Incubation was performed sequentially with beads functionalised with anti-EpCAM and anti-EGFR antibodies. To check epithelial origin and to discard blood cells, we performed an immunofluorescence assay on isolated cells in which pan-cytokeratin-positive; CD45-negative and nuclear stained (Hoechst) cells were considered as bona fide CTCs (Fig 5).

We conducted in parallel the quantification of CTCs isolated from patients by immunofluorescence and CellSearch^®^ analysis (Table 3). We detected the presence of CTCs (CK+; CD45-; nucleus) isolated with anti-EpCAM, anti-EGFR and/or anti-FGFR magnetic beads in 95.45% of the samples as compared to CellSearch^®^ data (in only one case we did not detect CTCs by our procedure while CellSearch^®^ did).

**Table 3:** Comparison of CTCs isolated by CellSearch or magnetic beads coated with anti-EpCAM, anti-EGFR or anti-FGFR from patients with metastatic cancer. Total refers to the sum of the three fractions.

Sample	TUMOR	CellSearch	EpCAM	EGFR	FGFR	Total
1	Colon	8	10	4	2	16
2	Colon	10	3	9	2	14
3	Colon	3	3	0	1	4
4	Colon	7	4	3	1	8
5	Colon	0	0	0	0	0
6	Colon	8	1	2	1	4
7	Colon	4	0	0	0	0
8	Prostate	6	9	9	1	19
9	Prostate	6	8	4	7	19
10	Prostate	1	4	1	1	6
11	Prostate	4	7	0	0	7
12	Breast	0	5	1	2	8
13	Breast	3	0	1	0	1
14	Breast	0	0	0	0	0
15	Breast	4	6	1	3	10
16	Endometrial	0	2	0	1	3
17	Endometrial	0	3	4	1	8
18	Endometrial	0	0	0	1	1
19	Endometrial	0	0	0	0	0
20	Endometrial	0	6	0	0	6
21	Endometrial	1	1	0	0	1
22	Endometrial	3	2	3	1	6

We isolated CTCs with anti-EpCAM magnetic beads with the same efficiency as CellSearch^®^ in 9.09% of the samples and with even higher efficiency in 45.45% of the cases (Table 3). The total amount of CTCs (isolated with EpCAM, EGFR and FGFR) was higher than that detected by CellSearch^®^in 68.18% of samples analysed with statistical significance (p = 0.0037). It is worth mentioning that, in the paired analysed samples by CellSearch^®^ and EpCAM fraction, there is no statistical significance (p = 0.73), showing the reliability of the proposed method.

We found the rate of CTCs in the EpCAM, EGFR and FGFR fractions highly in accordance with the results obtained with the cell lines. However, cell lines show 1–2 times higher expression of EpCAM and EGFR, considering all tumour types together or colon, breast and prostate tumoursindependently. The exception was the percentage of CTCs isolated with anti-EGFR in prostate tumourswhich was almost 4 times higher than that observed in the PC-3 cell line. Although FGFR isolated CTCs from the blood samples, it was less efficient than EpCAM or EGFR, which was in agreement with the analysedtumour cell lines.

**Fig 6:**
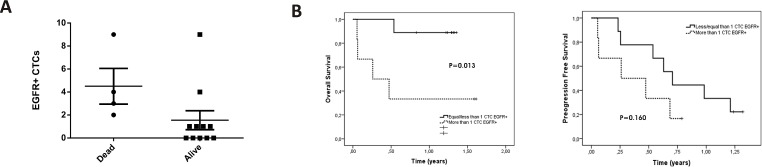
EGFR-positive CTCs correlates with worse prognosis. A) EGFR-positive CTC enumeration from patients with colon, prostate and breast metastatic cancer (from Table 3); (Spearman rank correlation coefficient = 0.575, p<0.05). B) Kaplan–Meier plot of overall survival or progression free survival based on EGFR-positive CTCs isolation.

Taking into account the cut-off of 5 CTCs in breast and prostate cancer and 3 CTCs in colon cancer which classifies patients with the worst prognosis using CellSearch^®^, there is an underestimation of CTC number in the majority of patients analysed when comparing total CTCs isolated with our coated magnetic beads. Although CellSearch is not validated for endometrial cancer analysis, we found CTCs in 6 of 7 patients while CellSearch only detected CTCs in 2 samples. Regarding clinical data, 5 of 22 patients included in this work died during this study, of which four(patient 4, 6, 8 and 9) had CTCs isolated with anti-EGFR (3, 2, 9 and 4 CTCs, respectively) while patient 11, who had no CTCs in the EGFR fraction, died from a tumour-independent cause. To check a possible correlation between the number of EGFR+ CTCs and worse prognosis, we performed a statistical analysis and found a positive correlation (Spearman coefficient = 0.575; *p* = 0.0249) (Fig 6A), not found for the EpCAM+ or FGFR+ CTCsfraction (data no shown). Kaplan–Meier analysis showed that higher levels of EGFR+ CTC (>1) were significantly associated with lower overall survival (Log Rank, p = 0.013) (Fig 6B). Moreover, progression free survival was also lower in the group of patients with more than 1 EGFR + CTCs, although does not reach statistical significance (Fig 6B). In addition, the presence of EpCAM + CTCs was associated with poor progression free survival in the analysed patients using coated magnetic beads (data no shown).

**Fig 7:**
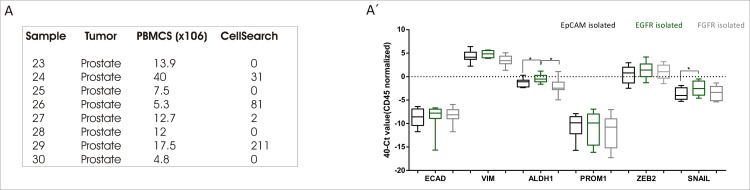
EGFR isolated CTCs depict differential subpopulation. Gene expression profiling of indicated genes in isolated CTCs from patients with metastatic prostate cancer using coated magnetic beads for the three fractions: EpCAM (inblack); EGFR (in green) and FGFR (in grey); (n = 8). CellSearch data regarding CTC number for each patient is summed in the box. Wilcoxon signed rank test, *p < 0.05.

To check if the isolated fractions represent tumoral subpopulations with differential phenotypes we performed gene expression analysis by RT-qPCR of CTCs isolated from patients with prostate metastatic cancer (n = 8; 26 ± 19.79 x 10^6^ PBMCs) using anti-EpCAM, anti-EGFR and anti-FGFR magnetic beads. The EGFR-isolated fraction expressed ALDH1 (p = 0.0391) and SNAIL (p = 0.0156) diferentiallycompared to the EpCAM fraction (Fig 7).

## Discussion

Carcinoma cells lose their epithelial features to change cell adhesion, activate proteolysis and to acquire motile properties for migration.Although a large number of cells enter the bloodstream in patients with cancer, only a small proportion of CTCs is able to survive, leave the bloodstream and colonise distant organs. Cells that undergo EMT have modified expression patterns of surface markers and currently platforms that capture CTCs do not cover all phenotypic heterogeneity of this tumour population[34, 35]. CTC detection relies so far on the epithelial characteristics of carcinoma cells. In fact, the CellSearch^®^ platform is only capable of capturing EpCAM-positive CTCs but not CTCs that are EpCAM-low or EpCAM-negative. EpCAM is used as an epithelial marker to capture CTCs; however, it has been described as having dynamic expression (may be downregulated or absent) associated with EpCAM loss during dissemination into the blood, cancer progression and metastasis [10, 36–38]. EpCAM has a dual role as a tumour suppressor gene or as an oncogene [39], and it has been proposed that epigenetic dysregulation could underlyieEpCAM expression. The phenotypic heterogeneity of EpCAM between epithelial tumours is well known, and it is found even among individual CTCs within the same sample[40]. Heterogeneity in EpCAM expression and the lack of cancer type-specific markers in most cancers limits CTC quantification. Thus, there is an urgent need for theinclusion of additional markers for optimising CTC detection methods. For example, Zhang and co-workers detected, isolated and characterisedEpCAM-negative CTCs present in the blood of patients with breast cancer [37]. They proved that current methods underestimate a CTC population with no EpCAM expression. Thus, the loss of EpCAM expression may explain the low CTC number in some patients with metastatic breast or prostate cancer, among other tumour types.

To recover CTCs with low or no EpCAM expression, we developed versatile magnetic beads. We demonstrated that using a combination of surface markers coated to the magnetic beads improves the recovery rates of spiked cells compared to EpCAM alone. We selected EGFR in addition to EpCAM to isolate CTCs; by using both markers (EpCAM and EGFR) we are able to cover all phenotypes in the selected cell lines.

This new approach resolves EpCAM dependency and encompasses a wide range of phenotypes, including mesenchymal features. It has been reported that cell lines with low EpCAM expression show low expression of other epithelial markers (cytokeratins 8, 18 and 19) or E-cadherin with high expression of vimentin[41]. Moreover, Punnose and colleagues reported that EGFR is highly expressed in low-EpCAM cells, thus suggesting that CTCs may be difficult to capture using a purely EpCAM-based capture mechanism. We confirm that EGFR is an alternative EpCAM-independent marker as we improved the recovery rates of the low EpCAM cell line MDA-MB-231. Moreover, we were able to efficiently isolate CTCs from patients with metastatic cancer with magnetic beads functionalised with anti-EGFR antibodies.

EGFR belongs to the HER/ErbB family of tyrosine kinase receptors. EGFR activation disrupts cell-cell adhesion by destabilizing the E-cadherin/β-catenin complex, promotes EMT and contributes to the acquisition of a motile phenotype, providing a link between oncogenic activation of these kinases and the induction of EMT [42]. EGFR function is frequently dysregulated in epithelial tumours, and EGFR signalling has been shown to play an important role both in cancer progression and in EMT-like transitions. Furthermore, the EGFR pathway controls proliferation, angiogenesis and apoptosis inhibition [43] and a statistically significant correlation between EGFR positive CTCs and cytokeratin-; vimentin+ and Slug+ CTCs has been reported, suggesting that CK- CTCs with higher EGFR expression reflect EMT [44].

Post-isolation protocols to identify CTCs are so far based on cytokeratin expression (mainly CK8/18/19), however, it seems clear that that during the progression of EMT, the down-regulation of EpCAM and CK is part of an oncogenic pathway that increases tumourinvasiveness and metastatic potential. Failure to detect CTCs or the existence of invisible CTCs due to thedownregulation or absence of CK have been published [45, 46]. It is therefore imperative to develop an alternative strategy aside from CK staining alone, regardless of the type and stage of cancer, to identify CTCs efficiently. The use of tissue specific or EMT markers could help to identify a higher number of CTCs.

It is worth mentioning that we detected several CK-; and CD45- cells when isolated with EpCAM, EGFR or FGFR; these cells had probably lost their epithelial phenotype and hence cytokeratin expression. It is likely that if these cells had been validated using other markers (mesenchymal or stemness) the total amount of detected CTCs would had been higher. Moreover, in some patient samples we found a significant amount of double-positive cells (CK+; CD45+), suggesting that there is a mixed population with unknown origin and clinical significance so far [47]. CTCs could have important crosstalk with the immune cells which explains the presence of both markers (CD45, CK) in some cells.

FGFR, the other marker used in this work for CTC isolation, plays a pivotal role in tumorigenesis due to the regulation of diverse processes such as cell survival, proliferation, inflammation, metastasis and angiogenesis [48]. A recent work demonstrated for the first time that the FGFR pathway regulates angiogenesis-dependent tumour growth [49]. In bladder cancer, BGJ-398 (an FGFR inhibitor) did not inhibit primary tumour growth but blocked the production of CTCs and the formation of lymph node and distant metastases in mice bearing orthotopically implanted mesenchymal UM-UC3 cells. Thus,FGFR1 is expressed by the mesenchymal subset of bladder cancer cells suggesting that the tumour EMT phenotype is an important determinant for the biological effects of FGFR inhibitors in patients[50]. Moreover, in aggressive prostate tumours, a novel molecular network, involving CRIPTO, AKT and FGFR signalling, was described in mesenchymal-like cancer cells[51]. All this suggests that FGFR could be a potential target for cancer treatment pointing to CTCs to avoid metastasis dissemination, but it is important to define the transcriptional targets responsible for mediating EMT.

We isolated low numbers of CTCs using anti-FGFR-coated magnetic beads from patient samples which correlates with the tumor cell line data. This minority subpopulation of CTCs could present a more mesenchymal phenotype and could be involved in the metastasis process[52–55]however, further studies must be done to confirm it. There is continuous change in both tumour and environmental influences that determines which cancer cell subpopulations are able to survive, proliferate, resist therapy or expand through the body. It has been postulated that each cell of a tumour is unique. In this sense, single-cell technology has been proposed as an alternative for identifying CTCs from enriched CTC samples [56–58].

In this work, we developed magnetic beads to isolate different subpopulations of CTCs from patients with metastatic cancer. It is worth mentioning that we can recoveredEpCAM-positive cells from patients samples as CellSearch did and at a higher ratio if the additional markers are considered. Taking into account that EpCAM-positive CTCs isolated from patients with metastatic cancer present a hybrid epithelial-mesenchymalphenotype [26, 59], we might suppose that an EpCAM-negative population will express EMT markers at a higher rate. In fact, we found overexpression of a stemness marker (ALDH1) and an EMT marker (SNAIL) in the EGFR fraction compared to the EpCAM fraction, confirming that our technology was able to isolate phenotypically different subpopulations of CTCs from the same patient. Moreover, although further monitoring of patients is required, there was a noticeable positive correlation between the number of EGFR+ CTCs and worse prognosis and overall survival, pointing to the EGFR+ subpopulation as more aggressive tumoral cells. Our findings suggest that sequential isolation of CTCs with EpCAM-independent markers improves subpopulation detection and reinforces the idea of the underestimation of the whole CTC population using only EpCAM-based isolation.

Metastatic cells could have lost their epithelial features, so it is necessary to incorporate other markers for CTC isolation and identification to avoid losing other subpopulations of CTCs. Functional assays could help to confirm CTC subpopulation features and the monitoring of patients could yield useful information of the prognostic value of these markers. Moreover, if the isolated CTC subpopulations grow *in vitro*, they could be tested for different proliferation or migration capabilities depending on their phenotype and allow the definition of molecular targets for personalised medicine and for *in vitro* monitoring of the therapy response. The other advantage of these magnetic beads is their versatility that permits the isolation of CTCs using other markers based on the tumour type and/or the molecular analysis of the primary tumour.

## Supporting Information

S1 Fig Flow cytometry analysis. **A)** Summary of Median Fluorescence Intensity of flow cytometer analysis of listed cell lines and markers. **B)** Flow cytometry analysis of SW480 and MDA-MB-231 cells incubated with anti-EpCAM (in blue), anti-EGFR (in orange) antibody or unlabelled cells (in red).
(TIF)

S1 Table Characteristics of human tumor cell lines. (DOC)

## References

[1] Hanahan D, Weinberg RA. Hallmarks of cancer: the next generation. Cell. 2011;144(5):646–74. Epub 2011/03/08. 10.1016/j.cell.2011.02.013 . 21376230

[2] Kang Y, Pantel K. Tumor cell dissemination: emerging biological insights from animal models and cancer patients. Cancer Cell. 2013;23(5):573–81. Epub 2013/05/18. 10.1016/j.ccr.2013.04.017 23680145 PMC3667710

[3] Harouaka R, Kang Z, Zheng SY, Cao L. Circulating tumor cells: advances in isolation and analysis, and challenges for clinical applications. Pharmacol Ther. 2014;141(2):209–21. Epub 2013/10/19. 10.1016/j.pharmthera.2013.10.004 24134902 PMC3947247

[4] Costa C, Abal M, Lopez-Lopez R, Muinelo-Romay L. Biosensors for the detection of circulating tumour cells. Sensors (Basel). 2014;14(3):4856–75. Epub 2014/03/13. 10.3390/s140304856 24618729 PMC4003971

[5] Ignatiadis M, Georgoulias V, Mavroudis D. Micrometastatic disease in breast cancer: clinical implications. Eur J Cancer. 2008;44(18):2726–36. Epub 2008/12/06. 10.1016/j.ejca.2008.09.033 . 19056036

[6] Lianidou ES, Markou A. Circulating tumor cells as emerging tumor biomarkers in breast cancer. Clin Chem Lab Med. 2011;49(10):1579–90. Epub 2011/08/02. 10.1515/cclm.2011.628 . 21801030

[7] Raimondi C, Gradilone A, Naso G, Cortesi E, Gazzaniga P. Clinical utility of circulating tumor cell counting through CellSearch((R)): the dilemma of a concept suspended in Limbo. Onco Targets Ther. 2014;7:619–25. Epub 2014/05/03. 10.2147/OTT.S46200 24790460 PMC4000244

[8] Gosens MJ, van Kempen LC, van de Velde CJ, van Krieken JH, Nagtegaal ID. Loss of membranous Ep-CAM in budding colorectal carcinoma cells. Mod Pathol. 2007;20(2):221–32. Epub 2007/03/16. 10.1038/modpathol.3800733 . 17361206

[9] Yanamoto S, Kawasaki G, Yoshitomi I, Iwamoto T, Hirata K, Mizuno A. Clinicopathologic significance of EpCAM expression in squamous cell carcinoma of the tongue and its possibility as a potential target for tongue cancer gene therapy. Oral Oncol. 2007;43(9):869–77. Epub 2007/01/09. 10.1016/j.oraloncology.2006.10.010 . 17207659

[10] Driemel C, Kremling H, Schumacher S, Will D, Wolters J, Lindenlauf N, et al Context-dependent adaption of EpCAM expression in early systemic esophageal cancer. Oncogene. 2014;33(41):4904–15. Epub 2013/10/22. 10.1038/onc.2013.441 . 24141784

[11] Bitting RL, Schaeffer D, Somarelli JA, Garcia-Blanco MA, Armstrong AJ. The role of epithelial plasticity in prostate cancer dissemination and treatment resistance. Cancer Metastasis Rev. 2014;33(2–3):441–68. Epub 2014/01/15. 10.1007/s10555-013-9483-z 24414193 PMC4230790

[12] Barriere G, Fici P, Gallerani G, Fabbri F, Zoli W, Rigaud M. Circulating tumor cells and epithelial, mesenchymal and stemness markers: characterization of cell subpopulations. Ann Transl Med. 2014;2(11):109 Epub 2014/12/10. 10.3978/j.issn.2305-5839.2014.10.04 25489583 PMC4245517

[13] Joosse SA, Gorges TM, Pantel K. Biology, detection, and clinical implications of circulating tumor cells. EMBO Mol Med. 2015;7(1):1–11. Epub 2014/11/16. 10.15252/emmm.201303698 25398926 PMC4309663

[14] Muinelo-Romay L, Vieito M, Abalo A, Nocelo MA, Baron F, Anido U, et al Evaluation of Circulating Tumor Cells and Related Events as Prognostic Factors and Surrogate Biomarkers in Advanced NSCLC Patients Receiving First-Line Systemic Treatment. Cancers (Basel). 2014;6(1):153–65. Epub 2014/01/24. 10.3390/cancers6010153 24452143 PMC3980598

[15] Gazzaniga P, de Berardinis E, Raimondi C, Gradilone A, Busetto GM, De Falco E, et al Circulating tumor cells detection has independent prognostic impact in high-risk non-muscle invasive bladder cancer. Int J Cancer. 2014;135(8):1978–82. Epub 2014/03/07. 10.1002/ijc.28830 . 24599551

[16] Cailleau R, Young R, Olive M, Reeves WJ Jr. Breast tumor cell lines from pleural effusions. J Natl Cancer Inst. 1974;53(3):661–74. Epub 1974/09/01. . 4412247 10.1093/jnci/53.3.661 PMC7364228

[17] Soule HD, Vazguez J, Long A, Albert S, Brennan M. A human cell line from a pleural effusion derived from a breast carcinoma. J Natl Cancer Inst. 1973;51(5):1409–16. Epub 1973/11/01. . 4357757 10.1093/jnci/51.5.1409

[18] Fogh J, Fogh JM, Orfeo T. One hundred and twenty-seven cultured human tumor cell lines producing tumors in nude mice. J Natl Cancer Inst. 1977;59(1):221–6. Epub 1977/07/01. . 327080 10.1093/jnci/59.1.221

[19] Giard DJ, Aaronson SA, Todaro GJ, Arnstein P, Kersey JH, Dosik H, et al In vitro cultivation of human tumors: establishment of cell lines derived from a series of solid tumors. J Natl Cancer Inst. 1973;51(5):1417–23. Epub 1973/11/01. . 4357758 10.1093/jnci/51.5.1417

[20] Kaighn ME, Narayan KS, Ohnuki Y, Lechner JF, Jones LW. Establishment and characterization of a human prostatic carcinoma cell line (PC-3). Invest Urol. 1979;17(1):16–23. Epub 1979/07/01. . 447482

[21] Gillis S, Watson J. Biochemical and biological characterization of lymphocyte regulatory molecules. V. Identification of an interleukin 2-producing human leukemia T cell line. J Exp Med. 1980;152(6):1709–19. Epub 1980/12/01. 10.1084/jem.152.6.1709 6778951 PMC2186024

[22] Tomlinson GE, Chen TT, Stastny VA, Virmani AK, Spillman MA, Tonk V, et al Characterization of a breast cancer cell line derived from a germ-line BRCA1 mutation carrier. Cancer Res. 1998;58(15):3237–42. Epub 1998/08/12. . 9699648

[23] Gazdar AF, Kurvari V, Virmani A, Gollahon L, Sakaguchi M, Westerfield M, et al Characterization of paired tumor and non-tumor cell lines established from patients with breast cancer. Int J Cancer. 1998;78(6):766–74. Epub 1998/12/02. 10.1002/(SICI)1097-0215(19981209)78:6<766::AID-IJC15>3.0.CO;2-L [pii]. . 9833771

[24] Colas E, Muinelo-Romay L, Alonso-Alconada L, Llaurado M, Monge M, Barbazan J, et al ETV5 cooperates with LPP as a sensor of extracellular signals and promotes EMT in endometrial carcinomas. Oncogene. 2012;31(45):4778–88. Epub 2012/01/24. 10.1038/onc.2011.632 . 22266854

[25] Monge M, Colas E, Doll A, Gonzalez M, Gil-Moreno A, Planaguma J, et al ERM/ETV5 up-regulation plays a role during myometrial infiltration through matrix metalloproteinase-2 activation in endometrial cancer. Cancer Res. 2007;67(14):6753–9. Epub 2007/07/20. 10.1158/0008-5472.CAN-06-4487 . 17638886

[26] Alonso-Alconada L, Muinelo-Romay L, Madissoo K, Diaz-Lopez A, Krakstad C, Trovik J, et al Molecular profiling of circulating tumor cells links plasticity to the metastatic process in endometrial cancer. Mol Cancer. 2014;13:223 Epub 2014/09/30. 10.1186/1476-4598-13-223 25261936 PMC4190574

[27] Ramírez LP, Landfester K. Magnetic Polystyrene Nanoparticles with a High Magnetite Content Obtained by Miniemulsion Processes. Macromolecular Chemistry and Physics. 2003;204(1):22–31. 10.1002. 10.1002/macp.200290052

[28] Vila A, Martins VC, Chícharo A, Rodríguez-Abreu C, Fernandes AC, Cardoso FA, et al Customized Desing of Magnetic Beads for Dynamic Magnetoresistive Cytometry. IEEE Transactions on Magnetrics. 2014;50(11):5101904 10.1109/TMAG.2014.2324411

[29] Hewitt RE, McMarlin A, Kleiner D, Wersto R, Martin P, Tsokos M, et al Validation of a model of colon cancer progression. J Pathol. 2000;192(4):446–54. Epub 2000/12/13. 10.1002/1096-9896(2000)9999:9999<::AID-PATH775>3.0.CO;2-K . 11113861

[30] Yin KB. The Mesenchymal-Like Phenotype of the MDA-MB-231 Cell Line. In: Gunduz M, editor. Breast Cancer—Focusing Tumor Microenvironment, Stem cells and Metastasis 2011 p. 386–402. 10.5772/20666

[31] Chen C, Zhao Z, Liu Y, Mu D. microRNA-99a is downregulated and promotes proliferation, migration and invasion in non-small cell lung cancer A549 and H1299 cells. Oncol Lett. 2015;9(3):1128–34. Epub 2015/02/11. 10.3892/ol.2015.2873 25663868 PMC4315021

[32] Toge M, Yokoyama S, Kato S, Sakurai H, Senda K, Doki Y, et al Critical contribution of MCL-1 in EMT-associated chemo-resistance in A549 non-small cell lung cancer. Int J Oncol. 2015;46(4):1844–8. Epub 2015/02/04. 10.3892/ijo.2015.2861 . 25647738

[33] Coey JMD. Magnetism and magnetic materials New York, NY: Cambridge Univ. Press 2010.

[34] Polioudaki H, Agelaki S, Chiotaki R, Politaki E, Mavroudis D, Matikas A, et al Variable expression levels of keratin and vimentin reveal differential EMT status of circulating tumor cells and correlation with clinical characteristics and outcome of patients with metastatic breast cancer. BMC Cancer. 2015;15(1):399 Epub 2015/05/13. 10.1186/s12885-015-1386-7 . 25962645 PMC4434869

[35] Yu M, Bardia A, Wittner BS, Stott SL, Smas ME, Ting DT, et al Circulating breast tumor cells exhibit dynamic changes in epithelial and mesenchymal composition. Science. 2013;339(6119):580–4. Epub 2013/02/02. 10.1126/science.1228522 23372014 PMC3760262

[36] Martowicz A, Spizzo G, Gastl G, Untergasser G. Phenotype-dependent effects of EpCAM expression on growth and invasion of human breast cancer cell lines. BMC Cancer. 2012;12:501 Epub 2012/11/01. 10.1186/1471-2407-12-501 23110550 PMC3519683

[37] Zhang L, Ridgway LD, Wetzel MD, Ngo J, Yin W, Kumar D, et al The identification and characterization of breast cancer CTCs competent for brain metastasis. Sci Transl Med. 2013;5(180):180ra48 Epub 2013/04/12. 10.1126/scitranslmed.3005109 23576814 PMC3863909

[38] Gires O, Stoecklein NH. Dynamic EpCAM expression on circulating and disseminating tumor cells: causes and consequences. Cell Mol Life Sci. 2014 Epub 2014/08/12. 10.1007/s00018-014-1693-1 . 25103341 PMC11113679

[39] van der Gun BT, Melchers LJ, Ruiters MH, de Leij LF, McLaughlin PM, Rots MG. EpCAM in carcinogenesis: the good, the bad or the ugly. Carcinogenesis. 2010;31(11):1913–21. Epub 2010/09/15. 10.1093/carcin/bgq187 . 20837599

[40] Sequist LV, Bell DW, Lynch TJ, Haber DA. Molecular predictors of response to epidermal growth factor receptor antagonists in non-small-cell lung cancer. J Clin Oncol. 2007;25(5):587–95. Epub 2007/02/10. 10.1200/JCO.2006.07.3585 . 17290067

[41] Punnoose EA, Atwal SK, Spoerke JM, Savage H, Pandita A, Yeh RF, et al Molecular biomarker analyses using circulating tumor cells. PLoS One. 2010;5(9):e12517 Epub 2010/09/15. 10.1371/journal.pone.0012517 20838621 PMC2935889

[42] Barr S, Thomson S, Buck E, Russo S, Petti F, Sujka-Kwok I, et al Bypassing cellular EGF receptor dependence through epithelial-to-mesenchymal-like transitions. Clin Exp Metastasis. 2008;25(6):685–93. Epub 2008/02/01. 10.1007/s10585-007-9121-7 18236164 PMC2471394

[43] Mitsudomi T, Yatabe Y. Epidermal growth factor receptor in relation to tumor development: EGFR gene and cancer. FEBS J. 2010;277(2):301–8. Epub 2009/11/20. 10.1111/j.1742-4658.2009.07448.x . 19922469

[44] Serrano MJ, Ortega FG, Alvarez-Cubero MJ, Nadal R, Sanchez-Rovira P, Salido M, et al EMT and EGFR in CTCs cytokeratin negative non-metastatic breast cancer. Oncotarget. 2014;5(17):7486–97. Epub 2014/10/04. 10.18632/oncotarget.2217 25277187 PMC4202138

[45] Mikolajczyk SD, Millar LS, Tsinberg P, Coutts SM, Zomorrodi M, Pham T, et al Detection of EpCAM-Negative and Cytokeratin-Negative Circulating Tumor Cells in Peripheral Blood. J Oncol. 2011;2011:252361 Epub 2011/05/18. 10.1155/2011/252361 21577258 PMC3090615

[46] Woelfle U, Sauter G, Santjer S, Brakenhoff R, Pantel K. Down-regulated expression of cytokeratin 18 promotes progression of human breast cancer. Clin Cancer Res. 2004;10(8):2670–4. Epub 2004/04/23. 10.1158/1078-0432.CCR-03-0114 . 15102669

[47] Lustberg MB, Balasubramanian P, Miller B, Garcia-Villa A, Deighan C, Wu Y, et al Heterogeneous atypical cell populations are present in blood of metastatic breast cancer patients. Breast Cancer Res. 2014;16(2):R23 Epub 2014/03/08. 10.1186/bcr3622 24602188 PMC4053256

[48] Feng S, Zhou L, Nice EC, Huang C. Fibroblast growth factor receptors: multifactorial-contributors to tumor initiation and progression. Histol Histopathol. 2015;30(1):13–31. Epub 2014/07/24. . 25053532 10.14670/HH-30.13

[49] Fons P, Gueguen-Dorbes G, Herault JP, Geronimi F, Tuyaret J, Frederique D, et al Tumor vasculature is regulated by FGF/FGFR signaling-mediated angiogenesis and bone marrow-derived cell recruitment: this mechanism is inhibited by SSR128129E, the first allosteric antagonist of FGFRs. J Cell Physiol. 2015;230(1):43–51. Epub 2014/04/25. 10.1002/jcp.24656 . 24760775

[50] Cheng T, Roth B, Choi W, Black PC, Dinney C, McConkey DJ. Fibroblast growth factor receptors-1 and -3 play distinct roles in the regulation of bladder cancer growth and metastasis: implications for therapeutic targeting. PLoS One. 2013;8(2):e57284 Epub 2013/03/08. 10.1371/journal.pone.0057284 23468956 PMC3582560

[51] Terry S, El-Sayed IY, Destouches D, Maille P, Nicolaiew N, Ploussard G, et al CRIPTO overexpression promotes mesenchymal differentiation in prostate carcinoma cells through parallel regulation of AKT and FGFR activities. Oncotarget. 2015;6(14):11994–2008. Epub 2015/01/19. 10.18632/oncotarget.2740 25596738 PMC4494918

[52] Bedussi F, Bottini A, Memo M, Fox SB, Sigala S, Generali D. Targeting fibroblast growth factor receptor in breast cancer: a promise or a pitfall? Expert Opin Ther Targets. 2014;18(6):665–78. Epub 2014/05/17. 10.1517/14728222.2014.898064 . 24833241

[53] Carnio S, Novello S, Bironzo P, Scagliotti GV. Moving from histological subtyping to molecular characterization: new treatment opportunities in advanced non-small-cell lung cancer. Expert Rev Anticancer Ther. 2014;14(12):1495–513. Epub 2014/09/04. 10.1586/14737140.2014.949245 . 25183305

[54] Ranieri D, Belleudi F, Magenta A, Torrisi MR. HPV16 E5 expression induces switching from FGFR2b to FGFR2c and epithelial-mesenchymal transition. Int J Cancer. 2015;137(1):61–72. Epub 2014/12/03. 10.1002/ijc.29373 . 25450802

[55] Zhu DY, Guo QS, Li YL, Cui B, Guo J, Liu JX, et al Twist1 correlates with poor differentiation and progression in gastric adenocarcinoma via elevation of FGFR2 expression. World J Gastroenterol. 2014;20(48):18306–15. Epub 2015/01/07. 10.3748/wjg.v20.i48.18306 25561797 PMC4277967

[56] Curtis C. Genomic profiling of breast cancers. Curr Opin Obstet Gynecol. 2015;27(1):34–9. Epub 2014/12/17. 10.1097/gco.0000000000000145 . 25502431 PMC4497788

[57] Fernandez SV, Bingham C, Fittipaldi P, Austin L, Palazzo J, Palmer G, et al TP53 mutations detected in circulating tumor cells present in the blood of metastatic triple negative breast cancer patients. Breast Cancer Res. 2014;16(5):445 Epub 2014/10/14. 10.1186/s13058-014-0445-3 25307991 PMC4303125

[58] Pestrin M, Salvianti F, Galardi F, De Luca F, Turner N, Malorni L, et al Heterogeneity of PIK3CA mutational status at the single cell level in circulating tumor cells from metastatic breast cancer patients. Mol Oncol. 2015;9(4):749–57. Epub 2014/12/30. 10.1016/j.molonc.2014.12.001 . 25539732 PMC5528771

[59] Barbazan J, Alonso-Alconada L, Muinelo-Romay L, Vieito M, Abalo A, Alonso-Nocelo M, et al Molecular characterization of circulating tumor cells in human metastatic colorectal cancer. PLoS One. 2012;7(7):e40476 Epub 2012/07/20. 10.1371/journal.pone.0040476 22811761 PMC3397799

