# Supplementary figures and images for: EGFR-Based Immunoisolation as a Recovery Target for Low-EpCAM CTC Subpopulation
Source: PLoS One. 2016 Oct 6;11(10):e0163705. doi: 10.1371/journal.pone.0163705 (PMC5053545; doi:10.1371/journal.pone.0163705)

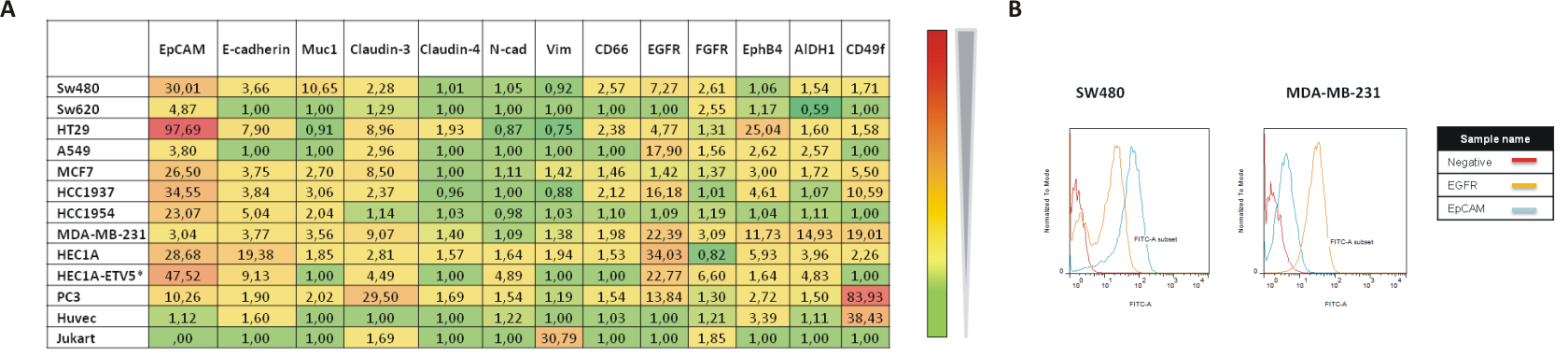

Supplement: S1 Fig — A) Summary of Median Fluorescence Intensity of flow cytometer analysis of listed cell lines and markers. B) Flow cytometry analysis of SW480 and MDA-MB-231 cells incubated with anti-EpCAM (in blue), anti-EGFR (in orange) antibody or unlabelled cells (in red). (TIF) [file pone.0163705.s001.tif]
