# Supplementary material for: EGFR-Based Immunoisolation as a Recovery Target for Low-EpCAM CTC Subpopulation
Source: PLoS One. 2016 Oct 6;11(10):e0163705. doi: 10.1371/journal.pone.0163705 (PMC5053545; doi:10.1371/journal.pone.0163705)
Supplement: S1 Table — (DOC) [file pone.0163705.s002.doc]

| **Cell line** | **Tumor type** | **Morphology** | **Disease** | **Origin** | **Mutations** | **Characteristics** |
| --- | --- | --- | --- | --- | --- | --- |
| **SW480** | Colon | Epithelial | Dukes' type B, Colorectal adenocarcinoma | Primary tumor | myc +; myb + ; ras +; fos +; sis +; p53 +; abl -; ros -; src - | EGF+ |
| **SW620** | Colon | Epithelial | Dukes' type C, colorectal adenocarcinoma | Derived from metastatic site: lymph node | myc +; myb + ; ras +; fos +; sis +; p53 +; abl -; ros -; src - | Derived from a metastasis of the same tumor as SW480. |
| **HT29** | Colon | Lymphoblast | Colorectal adenocarcinoma | Primary tumor | myc +; ras +; myb +; fos +; sis +; p53 +; abl -; ros -; src - |  |
| **A549** | Lung | Epithelial | Carcinoma | Primary tumor |  |  |
| **MCF7** | Breast | Epithelial | Adenocarcinoma | Derived from metastatic site: pleural effusion | WNT7B + | Estrogen receptor+ |
| **HCC1937** | Breast | Epithelial | TNM stage IIB, grade 3,primary ductal carcinoma | Primary tumor | BRCA1 (5382C mutation); her2/neu -, p53 - | Estrogen receptor -  Progesterone receptor- |
| **HCC1954** | Breast | Epithelial | TNM stage IIA, grade 3, ductal carcinoma | Primary tumor | her2/neu + (overexpressed) | Estrogen receptor -  Progesterone receptor- |
| **MDA-MB-231** | Breast | Epithelial | Adenocarcinoma | Derived from metastatic site: pleural effusion |  | EGF+  TGF+ |
| **HEC1A** | Endometrium | Epitelial | Stage IA endometrial adenocarcinoma | Primary tumor | c-fos + | Platelet activating factor (PAF)+ |
| **PC3** | Prostate | Epitelial | Grade IV, adenocarcinoma | Derived from bone metastasis |  |  |

**S1 Table. Characteristics of human tumor cell lines.**
